# Environmental conditions at the South Col of Mount Everest and their impact on hypoxia and hypothermia experienced by mountaineers

**DOI:** 10.1186/2046-7648-1-2

**Published:** 2012-09-04

**Authors:** Kent Moore, John Semple, Paolo Cristofanelli, Paolo Bonasoni, Paolo Stocchi

**Affiliations:** 1 Department of Physics, University of Toronto, Toronto, Ontario M5S 1A7, Canada; 2 Department of Surgery, University of Toronto, Toronto, Ontario M5S 1A7, Canada; 3 Institute of Atmospheric Sciences and Climate, National Research Council of Italy, Bologna, 40129, Italy; 4 EV-K2-CNR, Bergamo, 24126, Italy

**Keywords:** Hypoxia, Hypothermia, High-altitude conditions, Mount Everest

## Abstract

**Background:**

Hypoxia and hypothermia are acknowledged risk factors for those who venture into high-altitude regions. There is, however, little *in situ* data that can be used to quantify these risks. Here, we use 7 months of continuous meteorological data collected at the South Col of Mount Everest (elevation 7,896 m above sea level) to provide the first *in situ* characterization of these risks near the summit of Mount Everest.

**Methods:**

This is accomplished through the analysis of barometric pressure, temperature and wind speed data collected by an automatic weather station installed at the South Col. These data were also used as inputs to parameterizations of wind chill equivalent temperature (WCT) and facial frostbite time (FFT).

**Results:**

The meteorological data show clear evidence of seasonality, with evidence of pre-monsoon, monsoon and post-monsoon conditions. Low pressures, cold temperatures and high wind speeds characterize the pre- and post-monsoon periods with significant variability associated with the passage of large-scale weather systems. In contrast, the monsoon period is characterized by higher pressures, warmer temperatures and lower wind speeds with a pronounced reduction in variability. These environmental conditions are reflected in WCTs as low as −50°C and FFTs as short as 2 min during the pre- and post-monsoon periods. During the monsoon, the risk of cold injury is reduced with WCTs of order −20°C and FFTs longer than 60 min. The daily cycle in the various parameters is also investigated in order to assess the changes in conditions that would be experienced during a typical summit day. The post-monsoon period in particular shows a muted daily cycle in most parameters that is proposed to be the result of the random timing of large-scale weather systems.

**Conclusions:**

Our results provide the first *in situ* characterization of the risk of hypoxia and hypothermia on Mount Everest on daily, weekly and seasonal timescales, and provide additional confirmation as to the extreme environment experienced by those attempting to summit Mount Everest and other high Himalayan mountains.

## Background

The combination of low barometric pressure, cold temperatures and high winds that are common in high-altitude environments can compound hypoxic physiological stresses, resulting in an increased risk of hypothermia and frostbite [1-3]. Towards this end, a comprehensive analysis of mortality on Mount Everest indicated that hypoxia and hypothermia were the leading causes of nontraumatic fatalities amongst climbers, with approximately 30% of all fatalities being attributed to these factors [4]. There is also evidence to suggest that frostbite can inflict sherpas as well, for whom such cold injuries may be an occupational health issue related to inadequate information and clothing [5].

The impact that the low barometric pressure near the summit of Mount Everest has on success or failure on the mountain has long been a subject of interest amongst physiologists [3]. Messner and Habler's successful ascent of the mountain in 1978 without the use of supplementary oxygen provided the definitive answer to the question as to the ability of humans to survive in this hypoxic environment [3]. Notwithstanding this singular accomplishment, Mount Everest is so high that the low barometric pressure near its summit places humans exquisitely close to the tolerance limit for hypoxia, and changes in pressure near the summit as small as 4 hPa have been argued to be of physiological relevance [6,7]. Despite this sensitivity, there have been relatively few *in situ* measurements as to the summit barometric pressure and even fewer sequential *in situ* observations that would allow one to assess the impact that its temporal variability would have on the hypoxic stress experienced by climbers.

During the 1924 British Expedition to Mount Everest, daily barometric pressure measurements were made at Base Camp (5,029 m above sea level (a.s.l)) on the Tibetan side [8]. These data indicated that the summit attempt by Norton and Sommervell occurred during a period of higher barometric pressure, while that by Mallory and Irvine took place during a period of falling barometric pressure with the magnitude of the drop at Base Camp being approximately 18 hPa [9]. Subsequently, the barometric pressure drop at the summit was estimated to be 10 hPa, with the reduction in magnitude with height being a reflection of the vertical structure of the parent large-scale low-pressure system [10]. During the ‘Into Thin Air’ storm of May 1996, a summit barometric pressure drop of a similar magnitude was estimated to have occurred, based on global meteorological datasets [11].

The first *in situ* barometric pressure measurement at the summit of Mount Everest was made on 24 October 1981 when a value of 253 torr or 337 hPa was observed [12]. Another measurement was made on 23 May 1997 when a value of 259.5 torr or 346 hPa was recorded [13]. Subsequent re-calibration efforts suggested that the barometer used in 1997 was biased high at cold temperatures, and a corrected summit barometric pressure was estimated to be 252.5 torr or 337 hPa [13].^a^

In 1998, an automatic data recorder capable of measuring temperature and barometric pressure was placed on the South Col of Mount Everest (7,986 m a.s.l) [14]. The station operated from May 5 to August 26, although the temperature measurements after early July were unreliable as a result of snow covering the sensor [15]. The data showed a tendency for an increase in temperature and barometric pressure during the transition from the pre-monsoon to monsoon period [13,15]. In addition, a severe weather event characterized by high wind speeds that occurred on May 10 was captured by the device, and during this event, a barometric pressure drop of approximately 6 hPa was observed [15]. Using meteorological datasets, this event was shown to occur during the passage over Mount Everest of a jet streak, an embedded core of high wind speeds within the subtropical jet stream, that was associated with a tropopause fold, an intrusion of ozone-rich stratospheric air into the upper troposphere [15].

Comparisons of the barometric pressure and temperature as recorded by the device during 1998 were also made with the corresponding time series extracted from global meteorological datasets, and good overall agreement was found [15]. However, during the May 10 event, the global meteorological datasets underestimated the magnitude of the barometric pressure drop by approximately 40% [15].

The 1998 temperature data collected at the South Col have been used to estimate the wind chill temperature (WCT), defined as the temperature in still air that would result in the same steady-state facial heat loss as occurs at a given temperature and wind speed [16]. This work highlighted the extreme hypothermic stress experienced by climbers high on Mount Everest. However, the lack of *in situ* wind speed data required an assumption as to its value, limiting the usefulness of this initial estimate.

The first quantitative estimate of the risk of cold injury at high altitude has only recently been provided [17]. This was accomplished through the use of temperature and wind speed data extracted from global meteorological datasets of the sort that were validated previously with data from the Mount Everest region [15,18]. This study focused on two parameters: the WCT and the facial frostbite time (FFT), defined as the time it takes for facial flesh to freeze. Throughout the year near the summit of Mount Everest, it was shown that the typical WCTs and FFTs are always less than −30°C and 20 min, respectively [17]. During the spring climbing season, WCTs of −50°C and FFTs of 5 min are typical; while during severe storms, they can approach −60°C and 1 min, respectively. During the May 1996 ‘Into Thin Air’ storm, the instantaneous WCT and FFT approached values typically found during the winter months [17]. This work highlighted the crucial role that wind speed played in increasing the hypothermic stress near the summit and also confirmed that the previous estimate was biased low as a result of the assumption as to the magnitude of the wind speed in the region [16].

A retrospective analysis of the environmental conditions during the spring 2006 climbing season on Mount Everest, a season that was marked by two high-profile solo bivouacs above 8, 500 m [19], emphasized the important role that seasonality and variability associated with large-scale weather systems play in the hypothermic stress experienced by climbers [20]. In particular, the event early in May of that year, in which the climber died, occurred when hypothermic stress was approximately 1 standard deviation more severe than usual, while the event late in May, in which the climber survived, occurred when this stress was approximately 1 standard deviation less severe than usual [20]. In addition, the warming trend during May, when combined with a tendency for a reduction in wind speed during the month, also meant that the hypoxic and hypothermic stresses later in the month were less severe than those earlier in the month [20].

However, there have not been any estimates of the hypothermic stress high on the mountain that rely on *in situ* meteorological observations. In addition, the lack of a long time series of the background environmental fields has limited our ability to fully characterize the hypoxic stress near the summit and its impact on climbers. In this paper, we make use of a unique time series of barometric pressure, temperature and wind speed collected at the South Col of Mount Everest from May through December 2008 to characterize the environmental conditions high on Mount Everest and the resulting hypoxic and hypothermic stresses. In addition to being the highest elevation at which an automatic weather station has ever been deployed, the South Col is also important as it is the location of the high camp on the Nepalese side of the mountain, and hence, climbers typically spend up to 24 h at or above this location during their summit attempts.

## Methods

**Figure 1:**
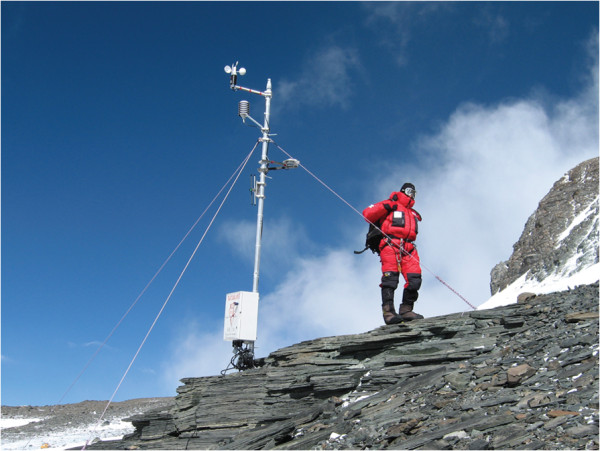
Automatic weather station as installed at the South Col of Mount Everest in May 2008.

On 13 May 2008, a team of Italian and Nepalese climbers, as part of the Stations at High Altitude for Research on the Environment Project succeeded in installing an automatic weather station at the South Col of Mount Everest (7,986 m a.s.l). This represents the highest automatic weather station anywhere in the world (Figure 1). The station was able to make continuous measurements of barometric pressure, temperature, wind speed and direction, relative humidity and solar radiation. Details on the instruments and level of the accuracy of the measurements are reported in Table 1. The station had a microwave downlink that allowed for real-time access to the data. The data were recorded every 10 minutes as average values, and for this paper, we use data over the period from May through December 2008. Due to the extreme environmental conditions of South Col and resultant malfunctioning of the meteorological instruments, the relative humidity measurements were terminated on 31 October, wind direction on 21 November and wind speed on 1 December 2008. Air temperature and barometric pressure measurements extended until 20 December and 17 December 2008, respectively.

**Table 1:** Instrumental setup of the automatic weather station installed at the South Col in May 2008

**Parameter**	**Instrument**	**Sensitive element**	**Range**	**Accuracy**	**Response time (s)**	**Sensor height (m)**
Air temperature	Thermohygrometer LSI Lastem DMA 672^a^	Pt100 Class B 1/3 DIN	−30°C to 70°C	±0.1°C (at 0°C)	10	2.5
Relative humidity	Thermohygrometer LSI Lastem DMA 672^a^	Capacitive hygrometer	0% to 100%	1.5% (from 5% to 95% at 23°C)	10	2.5
Barometric pressure	Vaisala PTB330^b^	BAROCAP© silicon capacitive absolute barometer	50 to 1,100 hPa	±0.75 hPa (from −40°C to +60°C)	1	2.5
Wind speed	LSI Lastem CombiSD DNA022^a^	three-cup and vane anemometer with optoelectronic disc	0 to 60 m/s	0.1 m/s + 1	2.5	3
Wind direction	LSI Lastem CombiSD DNA022^a^	2,000-Ω wire potentiometer	0° to 360°	1% full scale	0.74	3

^a^LSI Lastem, Milano, Italy. ^b^Vaisala Ltd., Suffolk, UK.

For this paper, the 10-min data were scanned for anomalous values and then averaged to provide hourly mean data. Data continuity was excellent, with good data available approximately 99% of the time. The hourly mean data were further averaged to provide daily mean values that were used in assessing the variability associated with large-scale weather systems and seasonality. In addition, the hourly mean data for each month were also averaged to provide information on the daily cycle. All times in this paper are with respect to Nepal Standard Time (NST), defined as GMT + 5:45 h.

Research into quantifying the combined impact of high wind speeds and low temperatures on hypothermic stress has been ongoing for over 60 years [21,22]. A recent collaboration between the American and Canadian Weather Services has resulted in the development of new expressions for WCT and FFT that combine results from wind tunnel studies of heat loss from human subjects with model studies of the heat transfer from the human face [23]. Please refer to Table 2 for the WCT and FFT formulae arising from this collaboration. We also introduce two new parameters: the wind chill temperature deficit (WCTD) that is defined as the difference between the observed temperature and the WCT, and the facial frostbite time deficit (FFTD) that is defined as the difference between the observed FFT and the FFT that would occur if the wind speed was zero. These new parameters provide an estimate of the additional hypothermic stress that is occurring as a result the combined effects of low temperatures and high wind speeds.

**Table 2:** **Equations used for the calculation of WCT and FFT**[23]

**Parameters**	**Equations**
Wind chill temperature	$\text{WCT}=13.12+0.621*T-11.37*V^{0.16}+0.3965*T*V^{0.16}$
Facial frostbite time	$\text{FFT}=(-24.5*(0.667*V+4.8)+2111)*{\left(-4.8-T\right)}^{-1.668}$

*V *is the wind speed (in kilometer per hour) and *T *is the air temperature (in degree Celsius). *FFT*, facial frostbite time; *WCT*, wind chill temperature.

It should be noted that the WCT is an equilibrium or steady-state parameter that does not take into account the dynamical aspects of hypothermia [24]. In addition, there may exist combinations of temperature and wind speed that provide the same WCT but different values of FFT [24]. It is for these reasons that it has been proposed that FFT, which is a dynamic parameter that is a measure of the time it takes for flesh to freeze, is a more robust indicator of the risk of cold injury [24].

In addition, it must be emphasized that these expressions were derived at sea level and there are meteorological and physiological issues with applying them at altitude [16,17]. In particular, the convective heat loss is proportional to the square root of the Reynolds number, which in turn depends on the product of the air density and wind speed [25]. The reduction in air density with height would result in a reduction in heat loss. However, both the WCT and FFT rely on the wind speed at a height of 10 m, and the expressions assume a decrease in wind speed with height in the lower 10 m of the atmosphere [23]. The measurements at the South Col were made at a height of 3 m (Table 1), and the scaling requires an effective increase of 50% in the wind speed in the expressions for WCT and FFT [23]. These two effects would cancel out to first order [17], and as a result, we have chosen to make no correction to the wind speed. In addition to these meteorological effects, the physiological effects of high altitude including hypoxia and dehydration can exacerbate the hypothermic stress as compared to sea level [2,26]. These effects are also not incorporated into the expressions. With the above caveats and in the absence of expressions for WCT and FFT that are appropriate for use at high altitude, it is our assertion that the existing expressions provide valid estimates of the risk for cold injury.

## Results

**Figure 2:**
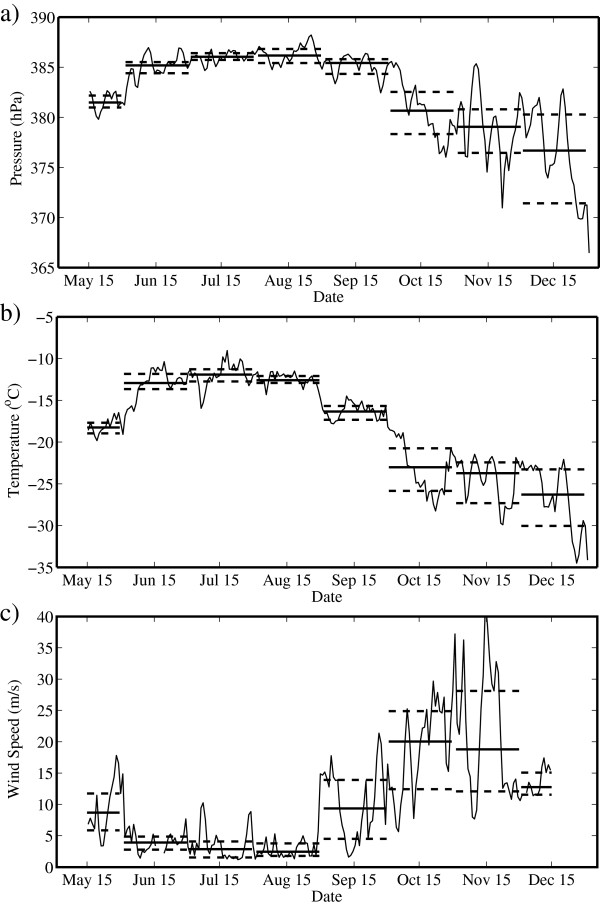
**Daily mean time series of (a) pressure, (b) temperature and (c) wind speed. **Measured at the South Col of Mount Everest from May to December 2008. The thick solid lines represent monthly mean values, while the thick dashed lines represent the first and third quartiles.

In Figure 2, we show the time series of the daily mean summit barometric pressure, temperature and wind speed from May through December 2008. Also shown are the median values as well as the first and third quartile values for each month. Table 3 provides the median and quartile values for each month. The median and quartiles were used rather than the mean and standard deviation to provide less emphasis on extreme events. All three time series clearly show the transitions between the pre-monsoon, monsoon and post-monsoon periods. These periods roughly correspond to the spring, summer and autumn seasons. It should be emphasized that the pre-monsoon period is not fully characterized as a result of the lack of data prior to mid May. The first transition, i.e., pre-monsoon to monsoon, occurred around June 1 and was characterized by an increase in pressure and temperature and a decrease in wind speed. For example, the median barometric pressure for May was 381.5 hPa and was 385.2 hPa during June. In addition, all three time series also show a decrease in variability across this transition. For example, the difference between the third and first quartile wind speed was 5.9 m/s during May and 2.1 m/s during June.

**Table 3:** Monthly median values of barometric pressure, temperature and wind speed at South Col during 2008

**Month**	**Median P**	**Delta P**	**Median T**	**Delta T**	**Median WS**	**Delta WS**
	**(hPa)**	**(hPa)**	**(°C)**	**(°C)**	**(m/s)**	**(m/s)**
May	381.5^a^	1.2^a^	−18.3^a^	1.3^a^	8.7^a^	5.9^a^
June	385.2	1.1	−12.9	1.8	3.9	2.1
July	386	0.7	−11.9	1.5	2.8	2.6
August	386.2	1.4	−12.6	0.8	2.4	2.0
September	385.4	1.5	−16.4	1.7	9.4	9.4
October	380.7	4.2	−23	5.1	20	12.4
November	379.1	4.4	−23.7	4.9	18.8	16
December	376.7	8.9	−26.3	6.8	12.8^a^	3.5^a^

Also shown are the differences between the third and first quartile values for barometric pressure (Delta P), temperature (Delta T) and wind speed (Delta WS). ^a^Values for which a full month's worth of data was unavailable.

Monsoon conditions, characterized by approximately constant median values and low variability, prevailed during the period from June through August. The transition to post-monsoon conditions occurred around the first of September and was characterized by decreasing barometric pressures and temperatures and increasing wind speeds as well as an increase in variability. For example, the median temperature was −12.6°C in August and decreased to −16.4°C in September, while the difference in the third and first quartile wind speeds increased from 2 m/s in August to 9.4 m/s in September.

The post-monsoon period was also characterized by the occurrence of numerous events in which there was a dramatic decrease in barometric pressure, including an event in early November when there was an approximate 10-hPa drop. These events, most likely associated with the passage of large-scale low-pressure systems, contributed to the large variability in barometric pressure during this period. For example, during October, the difference of the third and first quartile barometric pressure values was 4.2 hPa.

**Figure 3:**
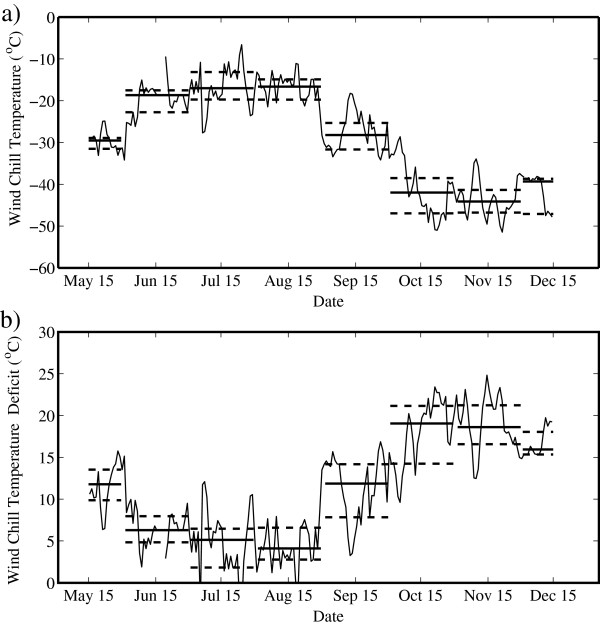
**Daily mean time series of (a) wind chill temperature and (b) wind chill temperature deficit. **Measured at the South Col of Mount Everest from May to December 2008. The thick solid lines represent monthly mean values, while the thick dashed lines represent the first and third quartiles.

Figure 3 shows the corresponding time series for WCT and WCTD. Recall that WCTD is the difference between the actual temperature and the WCT and so provides a measure of the impact that wind speed has on the rate of heat loss from the human body. Table 4 provides the median and quartile values for each month. The WCT time series has the same general shape as the temperature time series (Figure 1) but with a negative offset, illustrated by the WCTD time series, that varies from approximately 10°C in May to 5°C during June, July and August, and then from 15°C to 20°C from September through December. Both the WCT and WCTD show a decrease in variability during the monsoon period as compared to the pre- and post-monsoon periods.

**Table 4:** Monthly median values of wind chill equivalent temperature and wind chill equivalent temperature deficit

**Month**	**Median WCT**	**Delta WCT**	**Median WCTD**	**Delta WCTD**
	**(°C)**	**(°C)**	**(°C)**	**(°C)**
May	−29.5^a^	2.6^a^	11.8^a^	3.7^a^
June	−18.7	5.2	6.3	3.1
July	−17	6.6	5.1	4.6
August	−16.7	4.8	4.1	3.8
September	−28.2	6.4	11.9	6.3
October	−42	8.5	19	6.9
November	−44.1	5.4	18.6	4.6
December	−39.3^a^	8.4^a^	15.9^a^	2.7^a^

Measured at the South Col during 2008. Also shown are the differences between the third and first quartile values for wind chill equivalent temperature (Delta WCT) and wind chill equivalent temperature deficit (Delta WCTD). ^a^Values for which a full month's worth of data was unavailable.

**Figure 4:**
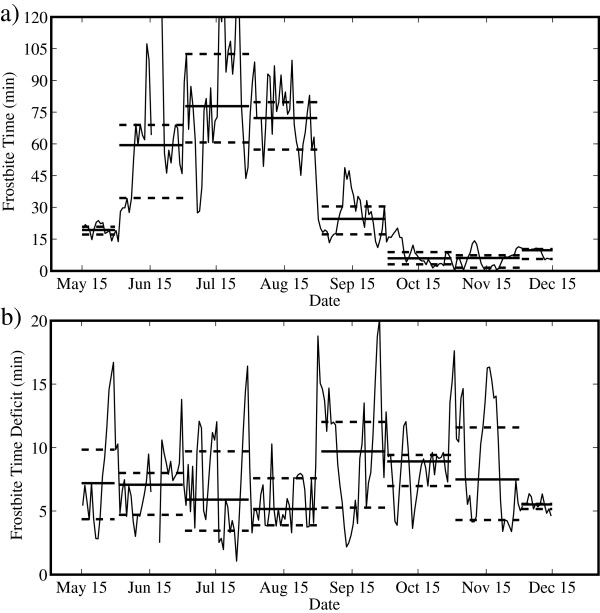
**Daily mean time series of (a) facial frostbite time and (b) facial frostbite time deficit. **Measured at the South Col of Mount Everest from May to December 2008. The thick solid lines represent monthly mean values, while the thick dashed lines represent the first and third quartiles.

Figure 4 shows the corresponding time series for FFT and FFTD. Again, recall that FFTD is the difference between the FFT and facial frostbite time in the case in which the wind speed is zero and so provides another estimate of the impact that wind speed has on heat loss from the human face. Table 5 provides the corresponding median and quartile values for each month. The FFT time series shows clear evidence of the pre-monsoon to monsoon transition as well as the monsoon to post-monsoon transition. For example, the median FFT is always less than 20 min during the pre- and post-monsoon periods, approaching 6 min in the post-monsoon period; while during the monsoon period, it is typically more than 60 min. The variability in FFT is also typically larger during the monsoon period as compared to the pre- and post-monsoon periods. The FFTD time series is approximately constant throughout the period from May to December at approximately 5 to 15 min. There are, however, periods where the FFTD is more than 15 min that correspond to situations where the wind speeds are high, as well as periods where FFTD is less than 5 min that correspond to situations where the wind speeds are low.

**Table 5:** Monthly median values of facial frostbite time and facial frostbite time deficit

**Month**	**Median FFT**	**Delta FFT**	**Median FFTD**	**Delta FFTD**
	**(min)**	**(min)**	**(min)**	**(min)**
May	19.3^a^	3.7^a^	7.2^a^	5.5^a^
June	59.4	34.5	7.5	4.1
July	77.2	41.8	14.5	infinity
August	72.2	22.4	8	11.9
September	24.5	13.2	9.7	6.8
October	5.9	5.7	8.9	2.5
November	6.1	5.9	7.5	7.3
December	9.7^a^	4.7^a^	5.5^a^	0.4^a^

Measured at the South Col during 2008. Also shown are the differences between the third and first quartile values for facial frostbite time (Delta FFT) and facial frostbite time deficit (Delta FFTD). ^a^Values for which a full month's worth of data was unavailable.

In addition to changes in the environmental, hypoxic and hypothermic risk parameters on monthly and seasonal timescales, climbers are also typically impacted by changes that occur during a typical day. This is especially true on summit day when climbers usually spend 24 to 36 h on the mountain at or above the South Col. Given the similarity noted above during each of the pre-monsoon, monsoon and post-monsoon periods, representative months during each period were selected for characterization of the daily cycle. May and October were selected for the pre- and post-monsoon periods since these months are when most of the summit attempts are made, with May being by far the month with the most attempts. Please note that, as discussed above, May is not fully characterized as a result of the lack of data during the first half of the month. July was selected to represent the monsoon period. Table 6 contains the daily mean values for these months as well as estimates of the variability in the amplitude of the daily cycle, as represented by twice the maximum of the standard deviation of the hourly data for each month. The use of median and quartile values generated similar results with evidence of higher noise levels due to the reduced number of degrees of freedom. It should be emphasized that the daily cycle as represented here includes the variability associated with passing large-scale weather systems. The timing of these systems is essentially random and so should average out with respect to the daily cycle. However, if the variability associated with these systems is large, then there will be a tendency for this variability to mask out any variability associated with the daily cycle. This is, of course, important for climbers as it implies that in this instance, extreme values can occur at any time during the day.

**Table 6:** Daily mean values of P, T, WS, WCT and FFT during May, July and October

	**May**	**July**	**October**
Mean P (hPa)	382.2	386.6	381.7
Delta P (hPa)	3.3	2.4	7.1
Mean T (°C)	−16.4	−9.3	−22.5
Delta T (°C)	5.5	9.6	7.7
Mean WS (m/s)	11.9	4.4	19.5
Delta WS (m/s)	16.1	7.8	19.7
Mean WCT (°C)	−28	−13.8	−40.1
Delta WCT (°C)	12.2	19.8	16.3
Mean FFT (min)	22.7	135.8	8.8
Delta FFT (min)	15.7	176.2	14.2

Also shown are estimates of the variability on the daily time scale, expressed as twice the standard deviation of the hourly values, of the various parameters. *FFT*, facial frostbite time; *P*, barometric pressure; *T*, temperature; *WCT*, wind chill equivalent temperature; *WS*, wind speed.

**Figure 5:**
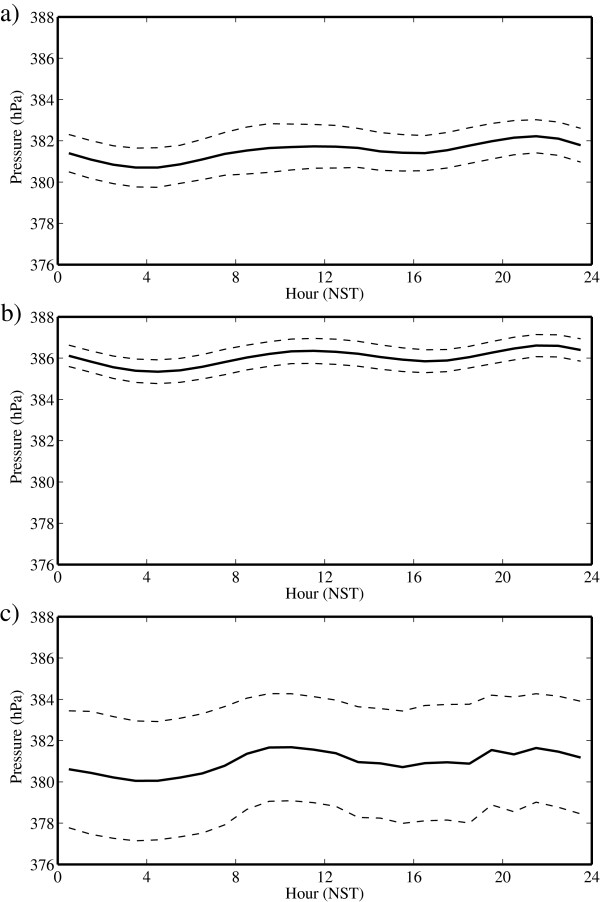
**Daily cycle of pressure at the South Col of Mount Everest. **During **(a) **May, **(b) **July and **(c) **October 2008. The dashed lines represent one standard deviation above and below the mean.

Figure 5 shows the daily cycle in barometric pressure during May, July and October with measures of the variability in the amplitude of the daily cycle. Again, the transition in barometric pressure across the periods is evident by the higher values during the monsoon period. In all months, the pressure has a semi-diurnal daily cycle with minima near 04:00 and 16:00 NST and maxima near 10:00 and 20:00 NST. The amplitude of the daily cycle is smallest during the monsoon period and largest during the post-monsoon period. Variability is also highest during the post-monsoon period, with a value in the order of 7 hPa. That is, on a given day in October, there is a 33% chance that the change in barometric pressure would be as large as 7 hPa.

**Figure 6:**
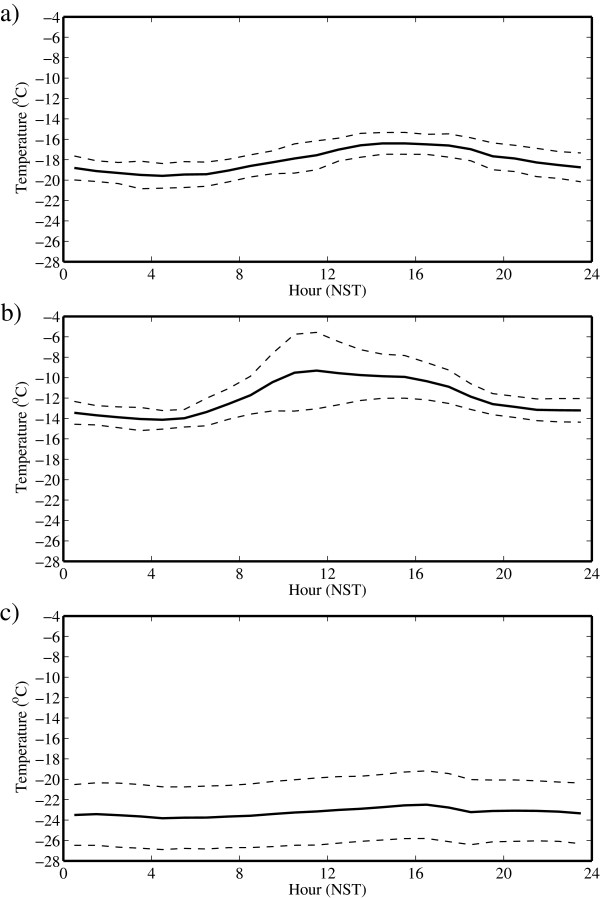
**Daily cycle of temperature at the South Col of Mount Everest. **During **(a) **May, **(b) **July and **(c) **October 2008. The dashed lines represent one standard deviation above and below the mean.

Figure 6 shows the corresponding time series for the temperature. There is evidence of a pronounced diurnal cycle in both May and July. This cycle is most pronounced in July as is the variability in the amplitude. In addition, the timing of the warmest temperatures occurs earlier in the day during July. In contrast, there is less evidence of a diurnal cycle during October. The variability in amplitude is nevertheless large.

**Figure 7:**
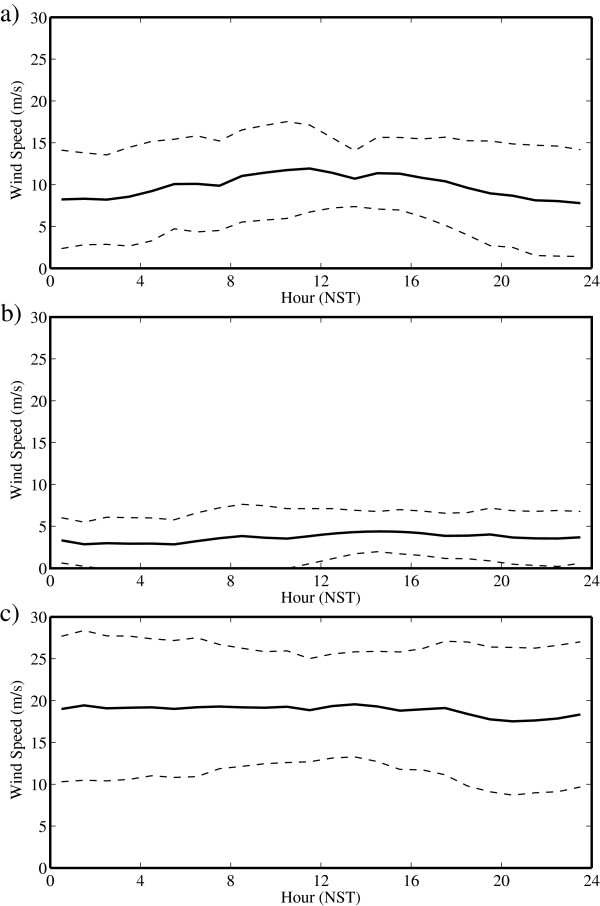
**Daily cycle of wind speed at the South Col of Mount Everest. **During **(a) **May, **(b) **July and **(c) **October 2008. The dashed lines represent one standard deviation above and below the mean.

Figure 7 shows the corresponding time series for wind speed. There is evidence of a diurnal cycle in wind speed during May, with the highest wind speeds typically occurring around 12:00 NST. During May, climbers could expect to experience an increase in wind speed as high as 10 m/s during the course of the day. In contrast, there is no evidence of a diurnal cycle during July or October. As expected from Figure 2, the wind speeds are generally weak during the monsoon period. Even though there is no evidence of a diurnal cycle during October, the average wind speed is largest at a value of 19.5 m/s.

**Figure 8:**
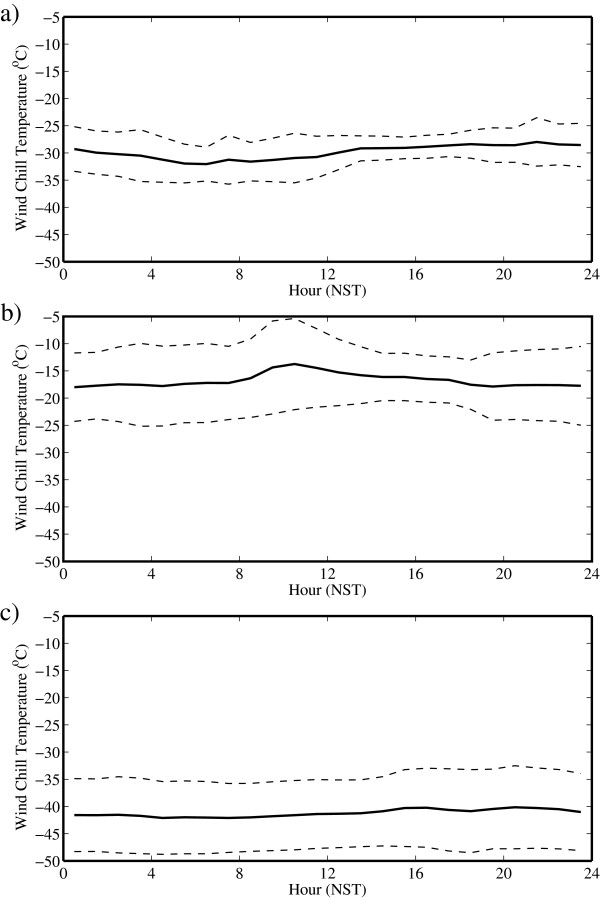
**Daily cycle of wind chill temperature at the South Col of Mount Everest. **During **(a) **May, **(b) **July and **(c) **October 2008. The dashed lines represent one standard deviation above and below the mean.

Figure 8 shows the corresponding time series for WCT. For this parameter, there is only a weak diurnal cycle for all the months, indicating that climbers would expect to experience the same hypothermic stress throughout the day. The reasons for this behavior are different for the various months. In May, the wind speeds are highest during the period of the day when the temperatures are warmest and *vice versa*. As a result, these two effects compensate, resulting in an approximately constant WCT throughout the day. Nevertheless, there is still the possibility of a large change in WCT that can exceed 20°C during the day in July. During July and October, the muted diurnal cycle in wind speed was most likely responsible for the lack of a pronounced daily cycle.

**Figure 9:**
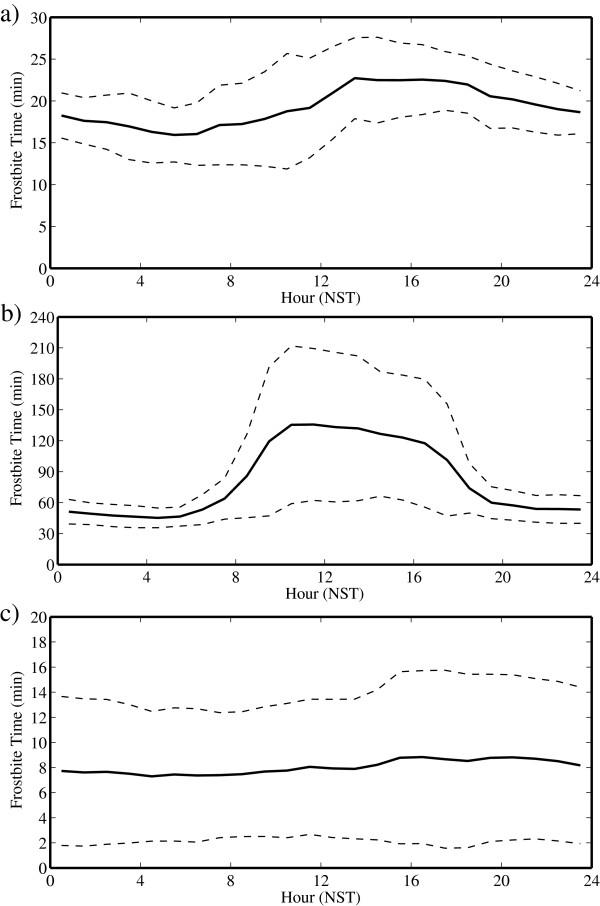
**Daily cycle of facial frostbite time at the South Col of Mount Everest. **During **(a) **May **(b) **July and **(c) **October 2008. The dashed lines represent one standard deviation above and below the mean. Note the change in the scale for the various sub-plots.

We conclude the presentation of the results with Figure 9, which shows the time series for FFT. In contrast to what occurred for WCT, there is evidence of a pronounced diurnal cycle in both May and July. This is most likely the result, as discussed above, of the difference between dynamic FFT parameter as compared to the more static WCT parameter. During May and July, the FFT time series closely resemble the temperature time series with minima in the early morning and maxima in the afternoon. It should, however, be noted that the FFTs during July are so high to be not a factor. In contrast, October again has little evidence of a diurnal cycle. During May, the average value of the FFT can be as long as 15 min for the whole day, while for October, it decreases to 8 min. Moreover, during October, the FFTs that can be lower than 2 min anytime during the day.

## Discussion

This paper represents the first comprehensive analysis of the *in situ* environmental conditions that occur high on Mount Everest and their impact on hypoxic and hypothermic stresses. This is accomplished as a result of the availability of high-frequency (i.e., less than 1 h) and long-term (i.e., over 7 months) barometric pressure, temperature and wind speed data from the South Col of Mount Everest. These data collected at an elevation of 7,986 m a.s.l, as well as estimates of the WCT and FFT that are derived from it, allow for the first characterization of variability due to large-scale weather systems and seasonality across the pre-monsoon, monsoon and post-monsoon periods. It also allows for a characterization of the daily cycle in these parameters for the same periods. It should be emphasized that the pre-monsoon period is not fully characterized as a result of the lack of data prior to the middle of May, when the station was installed during a climbing expedition. This is unfortunate as the pre-monsoon period is when most summit attempts occur.

As we have seen, seasonality plays an important role in the environmental, hypoxic and hypothermic conditions that climbers would experience on the mountain. Most noticeable is the impact that the progression in the seasons has on the conditions. During the spring climbing season, April is typically spent acclimatizing, and May is when most summit attempts occur. During the fall, the corresponding months are September and October. From the results presented, with the caveat noted above as conditions when we have no data during April and early May 2008, one can see that climbers in the spring are ascending into increasingly benign conditions, which are of course terminated by the onset of the monsoon in early June. The opposite is the case in the fall when climbers are ascending into increasingly severe conditions. In the fall, it is the cessation of the monsoon in early September that limits the date at which the process can begin.

The results presented in this paper confirm earlier work as to the extreme environment experienced by those attempting to summit Mount Everest and other high Himalayan mountains [16,17,20]. It has, however, the advantage of relying completely on *in situ* data and so is not subject to the caveats applied to earlier work that relied on global meteorological data or assumptions as to the character of some of the parameters. However, it should be noted that possible influence of local (complex) topography can affect meteorological observations (in particular the wind record), thus partially limiting the generalization of the obtained results to the entire region.

In addition, the results presented clearly show that the pre- and post-monsoon periods are characterized by an increase in variability on the weekly timescale that is undoubtedly the result of the passage of large-scale weather systems. During these months, large changes in barometric pressure, temperature and wind speed can occur on the weekly (or lesser) timescale. The magnitude of the barometric pressure changes during the pre- and post-monsoon periods are of sufficient magnitude to be of physiological importance [6,7]. This variability is also reflected in large variability in the hypothermic parameters. Thus, during these periods, care must be taken in timing ascents so as to minimize the severity of the environmental conditions. In contrast, the monsoon period is one in which the impact of large-scale weather systems is minimized, and this is reflected in the more uniform conditions during this period. Although conditions are generally more benign and stable, there are other hazards, such as heavy snowfall and avalanches, that assume a greater importance during this period.

The daily cycle in the various parameters was also investigated. A semi-diurnal cycle in barometric pressure was evident with an amplitude that was higher in the post-monsoon period. This semi-diurnal variability is most likely related to atmospheric tidal effects and is surprising in that data over the Tibetan Plateau suggest that a diurnal cycle in barometric pressure, forced by solar heating, should dominate [27]. This suggests that there may be some height-dependent effect with regard to the relative importance of the diurnal and semi-diurnal forcing of surface pressure variability.

A diurnal cycle in temperature was observed during both May and July. There was, however, a difference in that the warmest temperatures occurred earlier in the day during July. This is most likely the result of differences in the surface energy balance during the pre-monsoon and monsoon periods. During the pre-monsoon period, the South Col is snow-free, and hence, the high heat capacity of the surface would tend to delay the period of the warmest temperatures. In contrast, the South Col is usually snow covered in July, and so, the warmest temperatures would tend to occur around noon, following the daily cycle of solar radiation, as is observed. During October, there was evidence of a weak diurnal cycle in temperature that is in phase with that during May. Again, this is to be expected given the weaker solar forcing later in the year as well as a snow-free surface. The daily variability in temperature is, however, largest in October. This is most likely the result of increased large-scale weather activity whose timing of warm and cold advection is not tied to the solar forcing.

The wind speed shows a clear diurnal cycle in May, and it is most likely a signature of enhanced convective activity tied to solar forcing that is particularly evident in the development of strong thermal wind regimes along the Himalayan valleys located at lower altitudes [28]. Such convective activity can result in severe weather and even thunderstorms on the mountain [11]. The other months do not have as pronounced a diurnal cycle. During July, this is most likely the result of the generally weaker winds during this period, while in October, it is most likely the result of the aforementioned asynchronous timing of the large-scale forcing.

During May and October, the WCT and FFT are approximately constant throughout the day, indicating that the risk of hypothermia is essentially uniform. The reasons for this behavior are however different. During May, the wind speeds are highest during the day when the temperatures are warmest and *vice versa*. As a result, the wind speed and temperature effects cancel out. In contrast, during October, the wind speeds and temperatures are more uniform during the day. The daily variability in these parameters is highest in October, when it is possible anytime during the day to experience WCTs as low as −50°C and FFTs as short as 2 min. During July, the warmer temperatures and lower wind speeds result in a greatly reduced risk of hypothermia, with WCTs in the order of −20°C and FFTs in the order of 60 min. These results suggest that care must be taken throughout the summit day during the spring and fall climbing seasons, and even though temperatures may be warmer during the day, the higher wind speeds can nevertheless result in an elevated risk of hypothermia and frostbite.

Finally, the results presented provide one with a measure of the environmental risk factors at the South Col. However, wind speeds on the mountain typically increase with height as one moves closer to the core of the subtropical jet [11,15], and temperatures tend to decrease [10]. As a result, the approximate 1-km difference in height between the South Col and the summit can result in an elevated risk of hypothermia [17,20]. As a consequence, the values presented in this paper should be taken as lower bounds for the risk of cold injury above the South Col.

## Conclusions

Through the use of a unique set of meteorological observations from the South Col of Mount Everest, the world’s highest automatic weather station, we have provided the first *in situ* characterization of the risk of hypoxia and hypothermia on Mount Everest on daily, weekly and seasonal timescales. The results provide additional confirmation as to the extreme environment experienced by those attempting to summit Mount Everest and other high Himalayan mountains.

In the pre- and post-monsoon periods, the impact of large-scale weather systems can result in elevated hypoxic and hypothermic stresses as a result of lower barometric pressures, higher wind speeds and colder temperatures. In particular, the magnitude of the barometric pressure drops associated with these weather systems are of physiological significance [6,7] and occur on a timescale that does not allow for acclimation. The timing of these events is not tied to the daily cycle of solar radiation, and so, their impact can occur at any time during the day. This may complicate ascents on the mountain that are, for the most part, timed to allow for summiting early in the day.

Above the South Col, wind speeds tend to increase and temperatures tend to decrease [10,11]. This implies that the hypothermic stress above the South Col is higher than that described in this paper. This is in addition to the documented increase in the hypoxic stress above the South Col [3]. Thus, the results presented here represent lower bounds on the risks that occur at higher heights. Given that climbers may be spending more than 18 to 24 h above the South Col, these risks are significant [4].

Unfortunately, the results presented in this paper cannot address the important issue of inter-annual variability. This aspect of the meteorology of the high Himalaya has not been extensively studied. There is, however, evidence that large-scale meteorological processes such as El Niño can impact the weather on Mount Everest [10], thereby impacting the hypoxic and hypothermic stresses. Long-term trends arising from global warming can also impact these stresses through changes in the background meteorological fields [10,29,30].

## Endnotes

^a^In what follows, we will use hectopascal (hPa) as our unit for barometric pressure. The conversion to torr is accomplished by dividing by 1.333.

## Competing interests

The authors declare that they have no competing interests.

## Authors’ contributions

GWKM conceived the design of the study, participated in the data analysis and helped draft the manuscript. JLS participated in the design of the study and helped draft the manuscript. PC, PB and PS participated in data collection activities and helped draft the manuscript. All authors have read and approved the final manuscript.
